# Immunohistochemical expression of 8-oxo-7,8-dihydro-2′-deoxyguanosine in cytoplasm of tumour and adjacent normal mucosa cells in patients with colorectal cancer

**DOI:** 10.1186/s12957-015-0667-6

**Published:** 2015-08-07

**Authors:** Petar Matosevic, Tajana Klepac-Pulanic, Emil Kinda, Goran Augustin, Iva Brcic, Jasminka Jakic-Razumovic

**Affiliations:** Department of Surgery, University Hospital Center Zagreb, Kispaticeva 12, 10000 Zagreb, Croatia; Gynecology Department, Community Health Center Zagreb East, Vidriceva 38, 10000 Zagreb, Croatia; Department of Pathology, University Hospital Center Zagreb, Kispaticeva 12, 10000 Zagreb, Croatia

**Keywords:** 8-oxodG, Colorectal cancer, Stress, Oxidative, Survival, Colon

## Abstract

**Background:**

The aim of this research was to study the levels of 8-oxo-7,8-dihydro-2′-deoxyguanosine (8-oxodG) in tumour tissue samples of colorectal carcinoma based upon immunohistochemical detection and compare those results with patients’ outcome.

**Methods:**

Tumour blocks of patients surgically treated for colorectal cancer were evaluated by 8-oxodG immunohistochemical staining. The expression was analysed in 500 tumour cells. The percentage of positive cells, as well as staining intensity, was recorded, and Allred score was calculated. For each patient, data of age, gender, tumour size and location, margin status, histologic grade, tumour stage, lymph node status, vascular invasion, overall survival, and therapy protocols were collected. Tumour grade was divided into two groups as low and high grade.

**Results:**

In this study, 146 consecutive patients with primary colorectal carcinoma were included. All data were available for 138 patients, and they were included in this research. There were 83 male and 55 female patients; the median age was 64 years (range 35–87 years). The results showed shorter 5- and 10-year survival in patients with 8-oxodG positive tumour cells (5-year survival, *n* = 138, Mantel–Cox, chi-square 4.116, degree of freedom (*df*) = 1, *p* < 0.05; 10-year survival, *n* = 134, Mantel–Cox, chi-square 4.374, *df* = 1, *p* < 0.05). The results showed a positive correlation between Allred score and high tumour grade (two-tailed Spearman’s ρ 0.184; *p* < 0.05), as well as with non-polypoid tumour growth (two-tailed Spearman’s ρ 0.198; *p* < 0.05). There was no significant difference of 8-oxodG expression related to age, sex, blood group, size and tumour site, distance from the edge of the resected tumour margin, lymph nodes involvement, and vascular invasion.

**Conclusions:**

In this study, the positive correlation between 8-oxodG presence in the tumour cells, worse clinical outcome, higher tumour grade, and flat morphology was found.

## Background

Colorectal cancer (CRC) originates from the colonic mucosal epithelial cells and represents a genomic disease that is the result of the cumulative effect of external and hereditary factors. Progression of the tumour is due to the clonal cell expansion with a selective growth advantage compared to surrounding epithelial cells. It is well known that cells located at the bottom of the crypt are more susceptible to oxidative damage due to the presence of hydrogen peroxide, in relation to the well-differentiated surface cells [1]. These cells proliferate and often have a high metabolic index, and for that reason, deoxyribonucleic acid (DNA) is exposed to oxidative stress [2]. Abnormal accumulation of oxidative radicals underlies the pathology of a myriad of diseases and biological processes, such as ageing, the occurrence of neurodegenerative diseases, diabetes, atherosclerosis, asthma, cardiovascular disease, and various malignancies [3]. Since 8-oxo-7,8-dihydro-2′-deoxyguanosine (8-oxodG) was discovered in 1984, it is known in the literature as a marker of the deoxyguanosine damage and has been investigated in many studies as a potential marker of carcinogenesis [4]. From previous studies, it can be concluded that the presence of 8-oxodG has mutagenic potential in the modification of code in DNA replication, with consequent induction of guanine/cytosine to thymine/adenine (GC→TA) transversion mutation. Those lesions in the absence of repair become mutagenic and can lead to further accumulation of mutations and cancer [5, 6]. Most of the mutations present in sporadic CRC are the result of replacing the one base pair of DNA [7]. The presence of 8-oxodG in human DNA varies from 0.5 to 5 per 100,000 guanine bases and can induce damage to other bases of DNA [8]. The mechanism by which the accumulation of oxidative radicals contributes to pathological conditions includes damage or oxidative modification of biomolecules such as nucleotides, lipids, and proteins [9]. In patients with sporadic CRC, higher levels of whole-blood oxygen radical production during chemiluminescence were observed compared to healthy controls, although the difference was not found in patients with hereditary non-polyposis colorectal cancer (HNPCC) and familial adenomatous polyposis (FAP) syndrome. These findings suggest a potential role of oxygen free radical occurrence in sporadic CRCs [10]. Elevated levels of various markers of oxidative stress, such as 8-oxodG, nitrous oxide, lipid and glutathione peroxidase, catalase, and reduced levels of cytosine methylation in DNA were found in patients with CRC [11]. Among the techniques for determination of oxidative modification of nucleotides, methods such as high-performance liquid chromatography with the use of a mass spectrometer (HPLC-MS) and high-performance chromatography with the use of an electrochemical detector or gas chromatography with mass spectrometer results with quantified estimate. On the contrary, 8-oxodG monoclonal antibody immunohistochemical staining is a qualitative assessment, enabling analysis of the structural oxidative damage inside of the cell [12]. In this study, 8-oxodG immunohistochemical presence in colorectal carcinoma tumour cells was analysed, and results were correlated with multiple patient parameters and known CRC survival predicting factors.

The hypothesis of this study is higher levels of 8-oxo-7,8-dihydro-2′-deoxyguanosine (8-oxodG) in tumour tissue samples of colorectal carcinoma correlate with higher tumour grade and worse survival.

## Methods

### Design

The study was a retrospective cohort between 1 January 1999 and 31 December 2014 at the Department of Surgery University Hospital Center Zagreb, Croatia. The study was approved by the Ethics Committee of the University Hospital Center Zagreb and the Ethics Committee of the Medical School Zagreb, Croatia.

### Patients and sample

In this study, 138 tumour paraffin blocks were used from 138 eligible patients with primary colorectal carcinoma, out of 146 consecutive patients surgically treated at our institution between 1 January 1999 and 31 December 2000, excluding hereditary CRCs. During the surgery, presence of any distant metastases was recorded. Data of age, gender, location and size of tumour, tumour stage, histological grade, lymph node status, vascular invasion, distance from the nearest edge of resected margin, overall survival in months, and therapy protocols were collected for 146 patients, resulting in left censoring applied to 8 patients due to missing most of the data. The patients alive after 31 December 2014 were right censored at survival analysis. The survival status of 134 patients was available for 15 years of follow-up. According to histological tumour grade, the patients were divided into two groups: low grade (well- and moderately differentiated tumours) and high grade (poorly differentiated tumours).

### Immunohistochemistry

For diagnostic purposes after fixation, tumours were paraffin embedded, cut, and stained with a standard method (haematoxylin–eosin) for light microscope analysis. For additional immunohistochemical staining, 4-μm cuts were used. Primary antibody anti-8 hydroxyguanosine (N45.1, ab48508, Abcam, USA) was used for specimen staining using immunoperoxidase avidin–biotin method in an automatic stainer (Autostainer, Dako, Denmark). Positive control in immunohistochemical staining was epidermis from hairless mouse stained with this antibody. Negative control was the same tissue with avoiding primary antibody. The antigen–antibody reaction was visualized by 3,3-diaminobenzidine tetrahydrochloride (DAB).

### Evaluation of immunohistochemistry staining

**Fig. 1:**
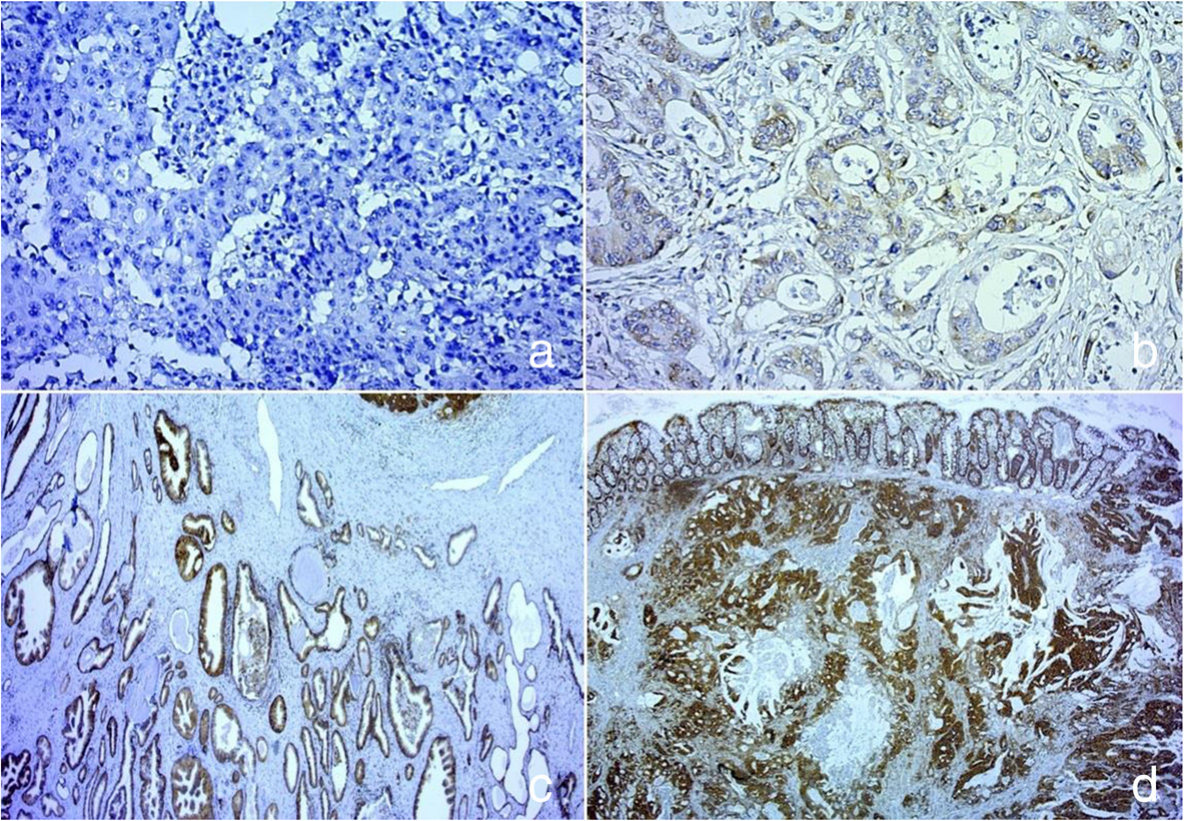
Intensity of antibody anti-8 hydroxyguanosine (N45.1, ab48508, Abcam, USA) staining is shown, classified into four grades as **a** negative, ×200; **b** weak, ×400; **c** medium, ×200; and **d** intensive, ×200. Counterstaining haematoxylin

The percentage of positive cells and staining intensity in a total of 500 counted tumour cells per patient sample were recorded. The intensity of staining was classified into four grades as negative (no staining), weak, medium, and intensive by observation under different microscopic magnification (Fig. 1). Allred immunohistochemistry score was calculated which is based on the percentage of cells that are stained by immunohistochemistry (0 to 5) and the intensity of that staining (0 to 3), resulting in a possible total score of 0–8 [13].

### Statistical analysis

All analyses were performed using SPSS for Windows version 22 (SPSS Inc, USA). The descriptive statistic was used. Difference between the groups was assessed using independent *t* test procedure and Pearson’s chi-square test where applicable. Survival was analysed using Cox regression models and Kaplan–Meier curves with starting point defined as the end of surgical treatment and end points defined as mortality at 15 years of follow-up. The Spearman’s test examined correlations and log-rank test examined statistical differences. Significance was determined by a probability value <0.05, with all *p* values two-sided.

## Results and discussion

### Results

**Fig. 2:**
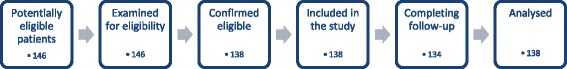
Flow diagram representing number of patients at each stage of the study

**Fig. 3:**
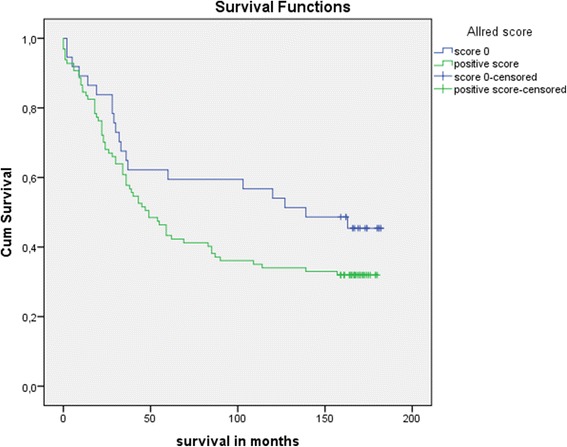
Kaplan–Meier curve, 15-year survival, starting at 100 % survival. *Green line* represents patients with cytoplasmic reaction for 8-oxodG in tumour cells and *blue line* represents patients without reaction. (5-year survival, n=138, Mantel–Cox, chi-square 4.116, df=1, p<0.05; 10-year survival, n=134, Mantel–Cox, chi-square 4.374, df=1, p<0.05)

**Fig. 4:**
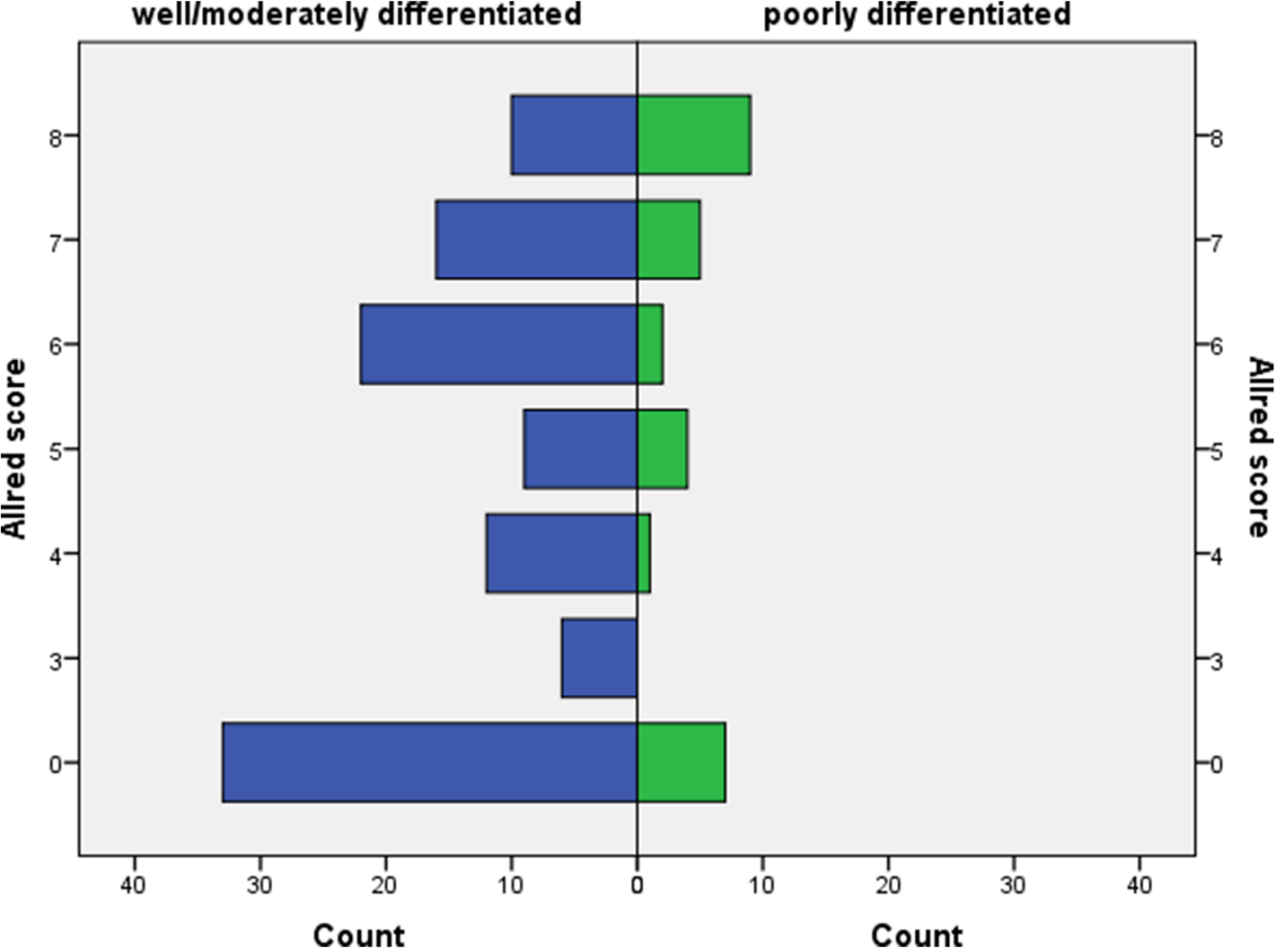
Case distribution through Allred score by tumour grade, well/moderately differentiated representing low-grade CRC tumours, and poorly differentiated representing high-grade tumours

**Fig. 5:**
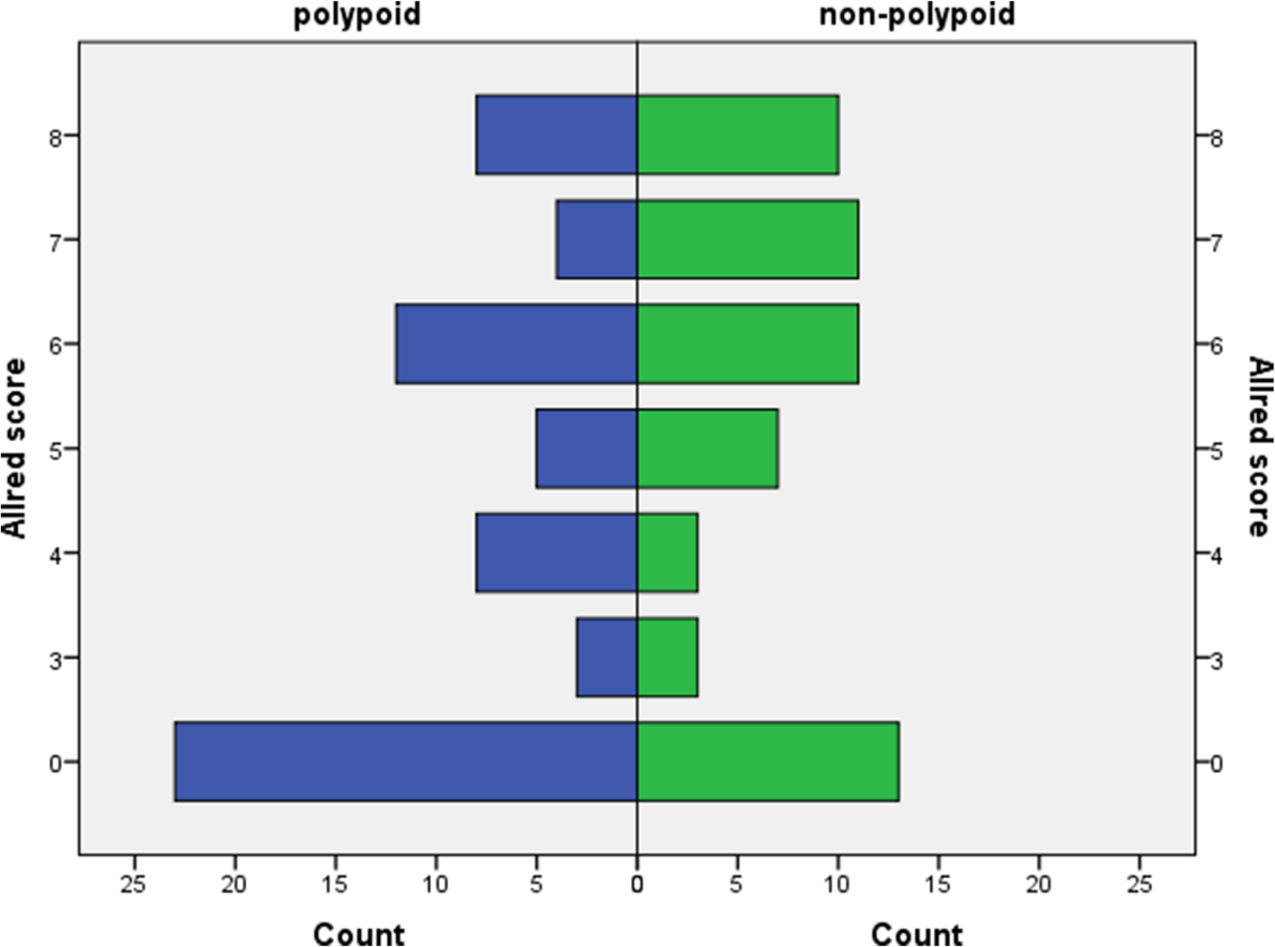
Case distribution through Allred score by macroscopic tumour appearance

Patient data and tumour characteristics are shown in Table 1, and flow diagram representing number of individuals at each stage of study is shown in Fig. 2. The median age of 138 CRC patients included in this study was 64 years (range 35–87 years). The studied group comprises of 55 women and 83 men. The majority of the patients were in higher local tumour stage (Dukes C in 46.7 % patients), and almost half of the patients were in clinical stages III and IV by tumour node metastasis (TNM) classification of malignant tumours from Union for International Cancer Control (UICC). Metastatic disease was found at the time of diagnosis in 15.2 % of the patients. None of the patients received neoadjuvant chemoradiotherapy preoperatively. Data on postoperative chemoradiotherapy is missing in 73 patients of 138 (52.9 %). Those patients were not excluded from the results analysis. Overall survival and 8-oxodG expression did not differ significantly compared to the type of chemoradiotherapy applied postoperatively. There was no difference in survival between the patients regarding circumferential resection margin. There were data about 5- and 10-year survival for 138 and 134 patients, respectively. The median follow-up was 169 months, range 166.75–171.25 months. During follow-up, 86 out of 134 patients died, 20 patients died in the group with Allred score 0, and 66 patients died in the group with positive Allred score. Negative correlation was noted between 5-year survival and intensity of 8-oxodG reaction in tumour cells (*N* = 138; ρ −0.191, *p* < 0.05), percentage of positive tumour cells (*N* = 138; ρ −0.151, *p* < 0.05), and Allred score (*N* = 138; ρ −0.157, *p* < 0.05). Shorter 5- and 10-year survival was noticed in the patients with 8-oxodG positive tumour cells (5-year survival, *n* = 138, Mantel–Cox, chi-square 4.116, degree of freedom (*df*) = 1, *p* < 0.05; 10-year survival, *n* = 134, Mantel–Cox, chi-square 4.374, *df* = 1, *p* < 0.05) (Fig. 3). Additionally, the results showed a positive correlation between higher Allred score and high tumour grade (two-tailed Spearman’s ρ, 184; *p* < 0.05) (Fig. 4). Also, higher Allred score correlated positively with non-polypoid tumour growth (two-tailed Spearman’s ρ, 198; *p* < 0.05) (Fig. 5).

**Table 1:** Clinical and tumour data of patients

	Colorectal cancer	Colorectal cancer	Allred score negative	Allred score positive	*p* value
	(*n* = 138)	(*n* = 134)	(*n* = 40)	(*n* = 98)	
Gender					0.087^c^
Females	55 (39.9 %)	55 (41 %)	20 (50 %)	35 (35.7 %)	
Males	83 (60.1 %)	79 (59 %)	20 (50 %)	63 (64.3 %)	
Average age (year)	64 SD ±10	64 SD ±10	65 SD ±10	64 SD ±10	0.662^d^
Dukes pathology					0.819^c^
A	30 (21.7 %)	29 (21.6 %)	10 (25 %)	20 (20.4 %)	
B	45 (32.6 %)	43 (32.1 %)	13 (32.5 %)	32 (32.7 %)	
C	63 (45.7 %)	62 (46.3 %)	17 (42.5 %)	46 (46.9 %)	
TNM clinical stage					0.929^c^
I	29 (21 %)	28 (20.9 %)	9 (22.5 %)	20 (20.4 %)	
II	43 (31.2 %)	41 (30.6 %)	12 (30 %)	31 (31.6 %)	
III	45 (32.6 %)	44 (32.8 %)	14 (35 %)	31 (31.6 %)	
IV	21 (15.2 %)	21 (15.7 %)	5 (12.5 %)	16 (16.4 %)	
Tumour grade (*n* = 136^a^; *n* = 132^b^)					0.373^c^
Low grade	108^a^ (79.4 %)	106^b^ (80.3 %)	33 (82.5 %)	75 (76.5 %)	
High grade	28^a^ (20.6 %)	26^b^ (19.7 %)	7 (17.5 %)	21 (21.4 %)	
Lymph node status					0.388^c^
Yes	63 (45.6 %)	62 (46.3 %)	17 (42.5 %)	46 (46.9 %)	
No	75 (54.4 %)	72 (53.7 %)	23 (57.5 %)	52 (53.1 %)	
Distant metastases					0.389^c^
Yes	21 (15.2 %)	21 (15.7 %)	5 (12.5 %)	16 (16.3 %)	
No	117 (84.8 %)	113 (84.3 %)	35 (87.5 %)	82 (83.7 %)	
Blood vessel invasion					0.106^c^
Yes	24 (17.4 %)	23 (17.2 %)	10 (25 %)	14 (14.3 %)	
No	114 (82.6 %)	111 (82.8 %)	30 (75 %)	84 (85.7 %)	
Survival (*n* = 134)					
5 years (median survival in months, 95 % CI, *N*° deaths)	‐‐	59 (95 % CI --; 69 deaths)	-- (14 deaths)	49 (95 % CI 32.9–65.1; 55 deaths)	0.083^e^
10 years (median survival in months, 95 % CI, *N*° deaths)	‐‐	59 (95 % CI 27.3–90.7; 80 deaths)	-- (95 % CI --; 16 deaths)	49 (95 % CI 32.9–65.1; 64 deaths)	0.036^e^
Median follow-up; range (months)	‐‐	169; 166.75–171.25; 86 deaths	n/a; 20 deaths	n/a; 66 deaths	0.111^e^
Allred score (8-oxodG)					n/a
0	40 (29 %)	37 (27.6 %)	40 (100 %)	0	
3	6 (4.3 %)	6 (4.5 %)	0	6 (6.1 %)	
4	13 (9.4 %)	13 (9.7 %)	0	13 (13.3 %)	
5	13 (9.4 %)	13 (9.7 %)	0	13 (13.3 %)	
6	24 (17.4 %)	24 (17.9 %)	0	24 (24.5 %)	
7	22 (15.9 %)	22 (16.4 %)	0	22 (22.4 %)	
8	20 (14.5 %)	19 (14.2 %)	0	20 (20.4 %)	

Statistical tests were conducted between Allred positive and negative patients

^a^In 5 year survival group 136 out of 138 patients had data about tumour grade

^b^In long-term survival group 132 out of 134 patients had data about tumour grade

^c^Pearson chi-square test for association

^d^Independent *t* test procedure was used, including data which were normally distributed and met criteria of homogeneity variances

^e^Log-rank)

Survival analysis (*n* = 134) was adjusted for age, gender, and tumour stage as confounders. Those confounders were identified to significantly impact survival estimated by Cox regression models as follows: age (*p* = 0.021; hazard ratio (HR) 1.028; 95 % confidence interval (CI) range 1.004–1.052), gender (*p* < 0.0001; HR 0.422; 95 % CI range 0.263–0.679), and TNM stage (stage I *p* < 0.0001; HR not applicable (n/a); 95 % CI range n/a; stage II *p* < 0.0001; HR 0.210; 95 % CI range 0.100–0.437; stage III *p* < 0.0001; HR 0.327; 95 % CI range 0.176–0.607; stage IV *p* = 0.024; HR 0.517; 95 % CI range 0.291–0.917). There was no difference in survival between the two groups of patients based upon Allred score negative versus positive status in tumour tissue and normal mucosis (Table 2).

**Table 2:** Unadjusted and adjusted survival

	*p* value	HR	95 % CI range
Unadjusted			
Allred score tumour tissue	0.116	0.669	0.405–1.104
Allred score normal mucosis	0.155	0.724	0.464–1.130
Adjusted			
Allred score tumour tissue	0.291	0.759	0.454–1.267
Allred score normal mucosis	0.214	0.748	0.474–1.182

Two groups of patients based upon Allred score negative versus positive status (both in tumour tissue and normal mucosis), assessed by unadjusted Cox regression model and adjusted for age, gender, and clinical stage

A positive correlation between the non-polypoid macroscopic appearance of tumours with a higher percentage of 8-oxodG positive tumour cells and higher intensity of reaction as well as higher Allred score was found (*n* = 138, two-tailed Spearman’s ρ 0.209; *p* < 0.05; ρ 0.189; *p* < 0.05; ρ 0.198; *p* < 0.05, respectively). There was no significant difference of 8-oxodG expression related to age, sex, blood group, size and tumour site, distance from the edge of the resected tumour margin, and lymph nodes involvement. There was no statistically significant correlation between the presence of 8-oxodG within the cytoplasm of tumour cells and the presence of vascular invasion.

### Discussion

8-oxodG as a marker of oxidative stress in cells can be evaluated via two groups of methods for 8-oxodG detection. Quantitative methods include high-performance liquid chromatography, gas chromatography–mass spectrometry, or enzyme-linked immunosorbent assay, while immunohistochemistry represents a qualitative method [12]. Qualitative methods allow morphological insight into the degree of oxidative DNA damage. Because of these characteristics, immunohistochemical detection methods with 8-oxodG specific monoclonal antibody are particularly suitable to assess the correlation between the 8-oxodG presence in tumour cells and cells of adjacent normal mucosa in the patients with CRC.

**Fig. 6:**
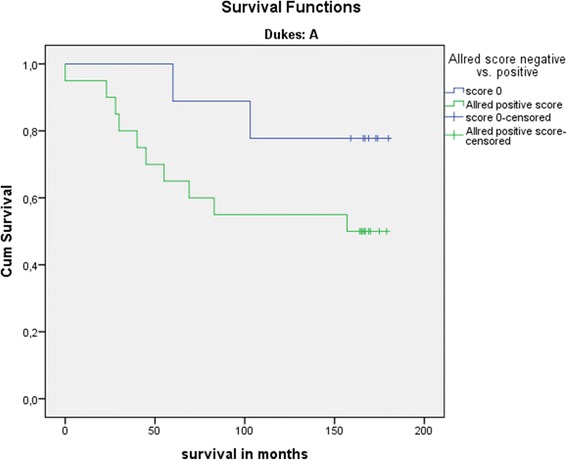
Kaplan–Meier curve, 15-year survival, Dukes A group, starting at 100 % survival. *Green line* represents patients with cytoplasmic reaction for 8-oxodG in tumour cells, *blue line* represents patients without reaction. Worse long-term survival of patients positive to 8-oxodG in early stage group is suspected due to systematic influence of oxidative stress

The problem of research studying the relationship between oxidative stress and carcinogenesis is the inability of a clear differentiation between oxidative damage as the cause or consequence [9]. Damage caused by oxidative stress present in a variety of chronic inflammatory conditions, and chronic diseases had been linked to carcinogenesis in many studies [14–16]. Our results showed that the group of patients who survive the first 5 years after surgery had a significantly lower percentage of 8-oxodG-positive tumour cells, lower intensity of the staining reactions, and consequently lower values by Allred score. One has to have in mind that the presented patients with CRC were at different stages of the disease during primary tumour resection, and their survival was influenced by a multitude of factors, including the various forms of the applied adjuvant oncological treatment, although none received neoadjuvant chemoradiotherapy that could theoretically alter oxidative status. Although there was no statistically significant difference in survival of the patients grouped by the local stage disease compared to 8-oxodG, long-term survival was better in the patients with early stage disease, such as Dukes A stage, suggesting that systemic influence of oxidative stress may predispose to worse prognosis (Fig. 6). Survival did not differ significantly between the groups of patients without reaction to 8-oxodG in tumour cells compared to the group of patients in whom there was a positive reaction to 8-oxodG. Ten-year survival confirmed the worse prognosis of the patients with higher intensity staining of 8-oxodG reaction, but a higher percentage of positive cells and higher Allred score did not result in a worse prognosis. The assumption is that patients with a positive reaction have higher clearance from survival pool, while influencing the survival curve in the long term. The assumption upon results from previous studies stated that the presence of 8-oxodG varied with the progression of the disease, and in patients with the development of distant metastases, stronger presence of 8-oxodG was noted [17].

The lack of 8-oxodG presence within the cytoplasm of tumour cells was associated with better overall outcome. Earlier publications described worse survival if 8-oxodG was present in renal cell carcinoma as well as in lung adenocarcinomas and non-small cell cancers [18, 19]. On the contrary, breast cancers immunohistochemically negative for 8-oxodG were associated with more aggressive disease and worse survival [20]. The above result attracts attention and requires further clarification. Earlier studies explored 8-oxodG presence in the tumour cell cytoplasm of patients without structural colon diseases and found no significant associations with age [17]. Hypothesis suggested that in patients without structural colon disease, cells with high levels of nuclear 8-oxodG undergo apoptosis due to detectable DNA damage, which is an indirect evidence of a functional mechanism to prevent further accumulation of mutations [17]. The patients who had failed the apoptosis of cells with damaged DNA have residual mutant phenotype, which may contribute to the further accumulation of mutations and the development of genetic instability [21]. A higher percentage of tumour cells with the reaction of 8-oxodG and higher Allred scores correlated positively with a less differentiated histological grade of the tumour. Based on these data, the assumption is that greater exposure to oxidative stress leads to the growth of cancers that are poorly differentiated, this alone influence survival with a worse prognosis [22].

Carcinogenesis of CRC favours adenomatous polyps as premalignant lesions, but non-polypoid adenomas (that include lesion slightly elevated above the mucosal lining of the plane or below the epithelium) are responsible for the formation of considerable number of CRCs. According to the data of Kudo et al. [23] on 14,014 adenomas and early CRCs, proportion of non-polypoid lesions was 44.5 %, and lesions located below the epithelium were present in 2.3 % of cases, but those were responsible for the occurrence of cancer in 32.4 % of cases [23]. It appears that greater exposure to oxidative stress contributes to non-polypoid cancer development. Given the relatively small sample size of this study, a definite conclusion could not be reached.

Vascular invasion is related to invasion of peritumoural vascular structures and is associated with poor prognosis of patients with CRC. More often it is the penetration through the wall of the vein responsible for the vascular invasion in colorectal carcinoma [24]. The assumption is that the pathogenesis of vascular invasion in CRC depends more on the invasiveness of tumour cells clones than to the exposure of tissues to oxidative stress. Data of vascular invasion did not have a significant impact on the results of this study.

## Conclusions

According to the previous research on the subject of oxidative stress and CRC and the results of this study, it seems that a certain part of the mutations responsible for CRC development may be attributable to the reactive chemical compounds. If the 8-oxodG-influenced CG-TA mutation results in the formation of CRC, these patients would have a worse prognosis. In later follow-up, patients with positive reaction to 8-oxodG are probably influenced more by general health impairment due to oxidative stress rather than the influence of oxidative stress on tumour carcinogenesis. The study results confirmed a positive correlation between level of 8-oxodG in tumour tissue of patients with CRC and higher tumour stage but failed to confirm worse outcome of such patients. The displayed results should be seen as a guideline for the proper formation of future prospective studies with a larger sample size to reach relevant information about oxidative stress influence on survival.

## Abbreviations

8-oxodG: 8-oxo-7,8-dihydro-2′-deoxyguanosine
CI: confidence interval
CRC: colorectal cancer
DNA: deoxyribonucleic acid
FAP: familial adenomatous polyposis
GC: guanine/cytosine
HNPCC: hereditary non-polyposis colorectal cancer
HPLC-MS: high-performance liquid chromatography with the use of a mass spectrometer
HR: hazard ratio
*N*: number
n/a: not applicable
*p* value: significance level of the test
ρ: Spearman’s rank correlation coefficient
TA: thymine/adenine
UICC: Union for International Cancer Control
